# Nurturing Grit among Medical Students

**DOI:** 10.12669/pjms.37.2.2999

**Published:** 2021

**Authors:** Tayyeba Iftikhar Mirza, Rahila Yasmeen, Usman Mahboob

**Affiliations:** 1 Dr. Tayyeba Iftikhar Mirza, MBBS, MHPE Department of Medical Education, Foundation University Medical College, Islamabad, Pakistan; 2 Dr. Rahila Yasmeen, BDS, DCPS- HPE, MHPE, PhD-HPE (Scholar) Department of Medical Education, Riphah International University, Islamabad, Pakistan; 3 Dr. Usman Mahboob, MBBS, MPH, FHEA, DHPE, Fellow FAIMER Institute of Health Professions Education and Research, Khyber Medical University, Peshawar, Pakistan

**Keywords:** Grit, Learning strategies, Medical students

## Abstract

**Objective:**

To identify the learning strategies used by the medical students with high Grit for design and implementation of a three months course, to assist the medical students having low Grit.

**Methods:**

A mixed-method study with explanatory sequential design was followed by an intervention to improve Grit amongst first year students at Foundation University Medical College from March to August 2019. Non-probability convenience sampling was used in the Phase-I. In Phase-II, through purposive sampling, students with high score on GRIT were interviewed. The interview questions were validated and piloted for clarity. All interviews were recorded, transcribed verbatim, and thematic analysis was done. The themes generated were used to design an intervention, which was implemented as a part of the curriculum for less Gritty students to see whether it can enhance Grit in them.

**Results:**

A total of 133 students participated out of which, 23(17%) had a high, whereas 10(7.5%) had a low level of Grit. Five themes generated from the transcripts, namely Planning, Metacognitive skills, Mastery learning, Cognitive strategies, and Self-regulation, that led the development of an intervention abbreviated as “RESET-P- GOALS”. A Wilcoxon signed-rank test showed that the intervention did elicit a statistically significant change in the Grit score in students having low Grit (Z = -2.8, p= 0.005).

**Conclusion:**

Good planning, Mastery learning and self-regulation are the reasons for success of Gritty students. The learning strategies with the name “RESET-P- GOALS” are effective in enhancing Grit in students with less score on Grit.

## INTRODUCTION

Grit is defined as “Perseverance and passion for long-term goals” that is working tirelessly towards challenges, maintaining effort and interest over years despite failure and hardships in progress.^1^ Grit is one of the most important non-cognitive skills that students can acquire in order to achieve long term goals, and opposing challenges and barriers s/he may face during education as well as professional life.^2^

The stress of complex medical courses, emotional immaturity, and adaptations to new surroundings are the challenges faced by the new medical entrants, although when they join the medical college they are full of hope, interest, creativity, and intelligence to make a difference.^3^ The non-conducive and competitive environment makes the students lose interest and their determination towards long term goals.^4^ This leads to early burnout, higher suicidal tendencies, feeling threatened from the success of others and ultimately easily give up and thus increase in dropout rate from medical colleges.^3^

Moreover, access to knowledge through information technology has changed the conventional teaching methods and it is now more important to provide guidance and support to help students in their learning.^5^ It is the need of an hour to teach our students the non-cognitive skills in order to meet the challenges. These skills improve the academic performance of students, and the students manage setbacks and stress in a better way.^6^ Grit is an important non-cognitive skill for facilitating positive youth development.^7^ Despite its importance, the literature lacks the grit-enhancing interventions which can be used to help students with low Grit.^8^

This study is a first step towards identifying the learning strategies used by the medical students with high Grit in order to design and implement a three months course, with the potential to assist the medical students having low Grit in order to reduce burnout, dropout, and suicidal tendency and make them better achievers.

## METHODS

A mixed-methods study with explanatory sequential three-step design was carried out over a period of six months (March-August 2019) Ethical approval was granted by Ethics Review Committee (Ref: Riphah/IIMC/ERC/19/0331, Dated: February 11, 2019), Islamic International Medical College, Rawalpindi and Ethical Review Committee (FF/FUMC/215-2/Phy/19, Dated: March 01, 2019) Foundation University Medical College, Islamabad.

### Participants

We invited the Year-1 MBBS students for participation in the study. Non-probability convenience sampling was used in the phase-I to segregate students with different level of Grit. A total of 133 students participated in Phase-I. In Phase-II, through purposive sampling, 22 students with a high level of Grit were interviewed to explore the learning strategies used by them. The second phase was an in-depth qualitative analysis, followed by developing a program and testing its hypothesis.

### Data collection and Analysis

To assess Grit, the twelve item self-report Grit scale^9^ was utilized. After a descriptive analysis of Phase I, we identified students with low, moderate and high levels of Grit. An interview guide was developed, having twelve open ended questions, which was validated by three medical education experts. Informed consent was taken. Semi-structured interview of Gritty students was conducted. Each interview lasted almost half an hour. All interviews were audio-recorded and transcribed verbatim. To enhance the credibility of results, data triangulation was done by using different sources of information. After ascertaining the accuracy of the interview transcripts, the methodological triangulation was done by comparing the results; the contents of the interviews were weighed against each other as well as the available literature. The interviews were also compared with the themes generated to further increase reliability. The themes that emerged after transcription and the data retrieved from literature, were used to design a three months course for students with a low level of Grit.

### Implementation of three months course

**Week 1:**

a) A session on “How to plan studies at Medical school.”

b) Each student was allocated a teacher mentor and a senior student mentor. This helped in personal and professional development of students and a support system.

**Week 2:**

Reflective writing through Gibbs Reflective cycle was introduced, and students were

asked to write reflective diaries on daily basis, and then plan accordingly. This helped student to become self-aware.

**Week 3:**

Awareness of learning style through VARK questionnaire. This activity was quiet useful as majority of students were not aware of their learning style.

**Week 4:**

Self-regulation through hands on activities like history taking and examination on a standardized patient, followed by constructive feedback. Encouragement and appreciation for their efforts, boosted their confidence and improved their self-efficacy.

**Week 5:**

Motivational Videos of successful individuals, helped in student engagement and building

of self-esteem in them.

**Week 6:**

Time management workshop helped them organize their activities according to importance.

**Week 7:**

Through exchange of written reflection, students discussed their experiences with each other. In this way there was collaborative social learning, and students felt they aren’t alone. This helped them persist.

**Week 8:**

Growth mindset and mindfulness workshop. To help students have faith in themselves, in order to put in effort and hard work and keep on rehearsing to achieve the goals.

**Week 9:**

Stress management workshop to cope up with the hardships.

**Week 10 & 11:**

Basic Life Support workshop to give them a sense of Responsibility.

**Week 12:**

### Metacognitive skills:

Students who use Metacognition efficiently are interested in learning activities, internally self-motivated and in habit of using goal setting, planning, and self-monitoring strategies.

## RESULTS

The results showed that amongst 133 students, 23(17%) students had a high level of Grit, 100(75%) had a moderate, and 10(7.5%) had a low level of Grit. Our study has explored the learning strategies used by students with high score on Grit for their academic success and to overcome hardships and setbacks. A total of five themes generated after the thematic analysis of interviews conducted with gritty students (Table-I).

**Table-I:** Themes generated from interviews with students with high score on Grit.

*Core theme*	*Subtheme*	*Representative quotes*
Planning	Strategic planning	I plan and allocate time for my studies. I try my level best to accomplish it in the allocated time.
Metacognitive skills	Mindfulness	I write diary every night, it’s totally a reflection of what I do..
Mastery learning	Deliberate practice	I study difficult concepts repeatedly; by breaking into chunks. I put lots of effort into difficult concepts ultimately I get hold of them.
Cognitive strategies	Study skills	I do collaborative study, I use social media and I watch videos. I believe one should do every task at its best.
Self regulation	Self awareness	Whenever I fail, I analyze the situation. Failures provide you an opportunity to learn and improve yourself.

**Fig.1:**
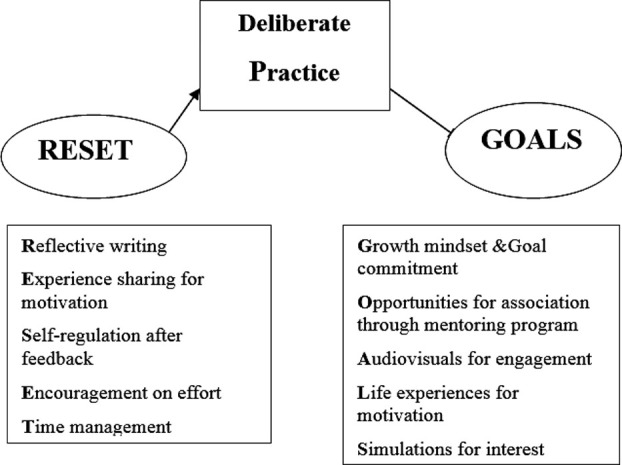
Teaching Strategies (RESET-P- GOALS).

The themes generated from interviews and construct of Grit available in literature were used to create a course with the name “RESET –P-GOALS” (Fig.1), through which students with the low Grit were taught for a period of three months.

The pre and post Grit score data were not normally distributed, so a non-parametric, Wilcoxon signed-rank test was used. The test showed that three months course (RESET-P- GOALS), did elicit a statistically significant change in Grit score (Z = -2.8, *p*= 0.005). The median score after the intervention (2.6) was greater than the median score before the intervention (2.2). The effect size calculated was (r= -0.6), signifying that the second mean (2.8±0.5) was greater than the first mean (2.1±0.3).

## DISCUSSION

This is the first study that aimed to explore the strategies used for learning and academic success by students with high score on Grit, in order to help students with low score on Grit. Students with high score on Grit showed high perseverance, through their optimistic attitude, which helps them to persist in all kinds of adversities. Students who show perseverance in their studies are more likely to achieve academic success.^10^ Gritty students were intrinsically motivated. According to self-determination theory, intrinsically motivated individuals have an internal locus of control, are determined to achieve, hunt for logical encouragement, and are excited about learning new things.^11^

Our study showed that interactive session, task-based learning, experience sharing and engaging the students through hands-on activities, helped them interact with one another and raised their level of interest and inculcated in them a sense of responsibility. Our Basic Life Support program was relevant to a real-world context, which contributed to attracting students’ attention and boosted their motivation. Reflection and timely feedback helped them to regulate themselves. Self-regulation helped them in goal setting, use of prior knowledge, progress monitoring, use of different learning strategies, and reflection. The incorporation of motivational talks and movies of real-time heroes encouraged the students and kept them motivated.

Previously, the effectiveness of interactive training programs in developing non-cognitive skills was studied. The findings revealed that interactive programs had helped foster the non-cognitive skills empathy, growth mindset, and Grit.^5^

The qualitative phase of our study signifies the importance of personal effort and deliberate practice towards achievement and better academic performance. Students with high score on Grit were more enthusiastic, regular in attendance and always ready to participate. One of the studies investigated the relationship between students’ perception of the link between personal effort and academic performance.^12^ The personal effort, characterized by the level of motivation, class attendance, taking an interest and being engaged in class. Results revealed that the grittier the participant, the more likely they were to connect their level of motivation to their academic performance.

Our study developed the program by utilizing the mediating variables and observable behaviors of students with high score on Grit that make them academically more successful, persistent and able to manage time efficiently. A study on elementary school students in which the curriculum included animated videos, mini case studies, and class activities like the importance of goal setting, constructive interpretation of setbacks and failures and plasticity of the human brain. It concluded that implementing this program they were able to foster Grit among their students in a classroom environment.^13^

Medical curriculum is the toughest among all other graduation programs; studies have proved that medical students are more prone to stress leading to depression.^14^ Our study signifies that students with high score on Grit realize the worth of life and its purpose, this helps them to remain positive and take setbacks as a challenge, and thus they are less prone to depression and burnout. A study conducted at a private medical college Faisalabad showed a negative correlation between Grit and depression and suggests to inculcate Grit in medical students to avoid depression.^15^ Association with others is an essential part of wellbeing.^16^ Students of first year were allocated senior student mentors and teacher mentors as well. Interaction with each other helped them in establishing a meaningful relationship. This helped in personal and professional development of students and a support system.

Our study and the literature suggest that both cognitive and non-cognitive factors need to interact for the learning to take place and to improve grit. Grit is all about the academic mindset, academic behavior, perseverance, persistence, effort, hard work, social skills and learning strategies which together lead to academic performance. The pre and post-test of Grit showed that Grit can be enhanced by teaching different strategies.

### Limitations of the study

The study is conducted in a single institute and on a single year students, thus limiting generalisability. It is a self-report questionnaire, so social desirability bias is the possibility, where respondents answer the question to show their thinking positive.

## CONCLUSION

The learning strategies with the name “RESET-P- GOALS’; are the practical tips for our educators. If every medical teachers attempt to incorporate Reflective writing, time management skills, Self-regulation through constructive feedback, Deliberate practice, Growth mindset exercises, regular sessions of motivational speakers, and Mentoring program, into their curriculum, we can assure that they are going to produce successful health professionals, with characteristics of perseverance, persistence, self-control and can face challenges with a Growth mindset. This course is the initial step towards helping our students to succeed. The Higher educational authorities should work in close collaboration with the teachers and design such strategies which will enhance students’ academic performance, which in turn will increase students’ persistence and retention. Medical colleges can use these interventions without investing many resources. These interventions will help reduce burnout, dropouts, suicidal rate, stress and anxiety among medical students, by inculcating in them passion, persistence, perseverance, and self-control; which will benefit the administration of college, staff, faculty and ultimately the community as a whole. Future research should involve multiple institutes so that the intervention (RESET-P- GOALS) can be applied to a larger sample of those individuals with less score on Grit to justify its significance.

### Authors’ Contribution:

**TIM**: conceived, designed, data collection, statistical analysis & manuscript writing.

**RY:** conceptualization, Methodology, review and final approval of manuscript.

**UM:** Initial development of proposal, multiple reviews, `critical analysis, editing of manuscript, and final approval of manuscript.

All authors are responsible and accountable for the accuracy and integrity of the work.
